# Long-term prognostic value of high-sensitivity cardiac troponin-I in patients with idiopathic dilated cardiomyopathy

**DOI:** 10.1515/med-2023-0837

**Published:** 2023-11-08

**Authors:** Yongchao Wang, Xiaolin Wang, Yulin Yang, Hao Xu, Jian Li

**Affiliations:** Department of Cardiology, The Affiliated Hospital of Qingdao University, Qingdao, 266000, China; Department of Cardiology, The Affiliated Hospital of Qingdao University, No. 1677 Wutaishan Road, Qingdao, 266000, China

**Keywords:** idiopathic dilated cardiomyopathy, high-sensitivity cardiac troponin-I, brain natriuretic peptide, long-term prognosis

## Abstract

Our objective was to evaluate the long-term prognostic value of high-sensitivity cardiac troponin-I (hs-cTn-I) in idiopathic dilated cardiomyopathy (DCM). First, patients were divided into an end-event group (*n* = 55) and a non-end-event group (*n* = 67). Then, patients were included in the subgroup analysis to compare the diagnostic value of brain natriuretic peptide (BNP) and hs-cTn-I in different populations. hs-cTn-I and BNP concentrations were higher in the end-event group. The Cox regression analysis indicated that high hs-cTn-I was a risk factor for poor long-term prognosis. Receiver operating characteristic analysis showed that the area under the curve (AUC) for hs-cTn-I to predict end events was 0.751, and the AUC for BNP was 0.742. The correlation analysis suggested that hs-cTn-I was related to the percentage change in left ventricular internal diameter at end-diastolic and left ventricular ejection fraction. Subgroup analysis showed that compared with BNP, hs-cTn-I was more suitable for predicting end events in patients with preserved renal function (AUC: 0.853 vs 0.712, *P* = 0.04). In conclusion, hs-cTn-I is a potential biomarker for evaluating long-term prognosis in idiopathic DCM, and its predictive value is higher than that of BNP in patients with preserved renal function.

## 1 Introduction

Idiopathic dilated cardiomyopathy (DCM) is defined as left ventricular or biventricular dilation and systolic dysfunction in the absence of an abnormal load (hypertension and vascular disease) or coronary artery disease sufficient to cause global systolic impairment according to the recommendations for classifying cardiomyopathies [1]. DCM is the main cause of heart failure with a reduced ejection fraction and the main indication for heart transplantation worldwide [2]. Troponin-I has been demonstrated to be related to all-cause mortality [3], heart transplantation, and the clinical need for an automatic implantable cardioverter defibrillator [4] in individuals with idiopathic DCM. Brain natriuretic peptide (BNP) is widely used for the diagnosis [5] and prognosis [6] of heart failure, the symptom of idiopathic DCM in the end stage [7]. Studies have also indicated a strong association of high-sensitivity cardiac troponin-I (hs-cTn-I) with the risk of new-onset heart failure [8]. However, there is currently a lack of studies directly comparing the ability of hs-cTn-I and BNP to predict long-term adverse outcomes, such as heart failure and cardiac death, in patients with idiopathic DCM. This study aimed to clarify the correlations among hs-cTn-I, BNP, and the long-term prognosis of patients with idiopathic DCM to provide a method for the clinical evaluation of long-term prognosis in patients with idiopathic DCM.

## 2 Subjects and methods

### 2.1 Subjects

This study was a retrospective study. Patients over 18 years of age diagnosed with idiopathic DCM at The Affiliated Hospital of Qingdao University from January 2020 to December 2021 were included in this study. Diagnosis referred to the recommendations for classifying cardiomyopathies [1]. The exclusion criteria were as follows: (1) severe valve regurgitation or stenosis; (2) uncontrolled hypertension (average blood pressure exceeding 160/100 mmHg) for more than 2 years; (3) patients with a history of drinking alcohol with a daily alcohol intake exceeding 125 mL for more than 10 years; (4) patients with a previous diagnosis of myocarditis; (5) patients with previous diagnosis of malignancy; (6) patients with end-stage heart failure which was defined as New York Heart Association class Ⅲ–Ⅳ, despite optimal medical and device therapy, intolerance or withdrawal of evidence-based heart failure medications such as beta blockers, angiotensin converting enzyme inhibitors due to hypotension, diuretic-refractory volume overload, or need for intravenous inotrope; (7) patients with severe liver and kidney dysfunction, where severe hepatic dysfunction was defined as an increase in bilirubin that exceeded 10 times the normal value, prothrombin activity <40%, or the presence of hepatic encephalopathy, and severe renal insufficiency was defined as the estimated glomerular filtration rate less than 60 mL/min; (8) patients with elevated hs-cTn-I that can be explained by myocardial infarction, aortic disease, myocarditis, or other heart diseases; and (9) patients with ischemic heart disease. All patients underwent coronary artery CT angiography or coronary angiography. Patients with stenosis of more than 50% in one or more main coronary arteries (right coronary artery, left main coronary artery, left anterior descending artery, left circumflex artery) or stenosis of more than 50% in two or more branch coronary arteries were excluded from the study. A total of 122 patients were enrolled in the study.

### 2.2 Data collection

Clinical data of the patients, including sex, age, past medical history, diagnosis, and treatment process, were obtained by consulting the electronic medical records. At the end of the follow-up period, all patients underwent cardiac ultrasonography a second time to evaluate the development of idiopathic DCM. This study has been approved by the Ethics Committee of The Affiliated Hospital of Qingdao University (QYFY WZLL 26432).

### 2.3 Study endpoint

The primary endpoint of this study was a composite endpoint consisting of (1) death due to DCM during the follow-up period; (2) readmission or emergency visit due to acute or chronic heart failure confirmed by symptoms, objective evidence, and biomarkers, such as BNP, while ruling out other causes of heart failure, such as myocardial infarction and infection, during the follow-up period; and (3) new-onset arrhythmias (e.g., atrial fibrillation). The definition of endpoint referenced 2017 cardiovascular and stroke endpoint definitions for clinical trials [9].

### 2.4 Research grouping

According to whether they had an end event within the follow-up period, the patients were split into two groups: the end-event group (*n* = 55) and the non-end-event group (*n* = 67). According to the estimated glomerular filtration rate (more than 60 mL/min or not), sex, age (greater than 60 years or not), and whether there was diabetes mellitus, patients were included in a subgroup analysis to assess the efficacy of hs-cTn-I and BNP in predicting the long-term prognosis in patients with idiopathic DCM.

### 2.5 Statistical processing

SPSS 22.0 software was used for statistical analysis. Measurement data conforming to a normal distribution are expressed herein as mean ± standard deviation (SD), and those conforming to a skewed distribution are expressed as *Q*_1_(*Q*_2_, *Q*_3_). Measurement data with a normal distribution and uniform variance were analyzed by independent sample *t* tests, and those with a skewed distribution or uneven variance were analyzed by nonparametric tests. Count data are expressed herein as frequencies or percentages, and comparisons between groups were made by Pearson’s chi-square tests. Clinical characteristics were all initially tested with univariate Cox regression analysis, and all variables with a value of <0.1 were considered for inclusion in the multivariate model. The association between hs-cTn-I, BNP, and percentage changes in cardiac ultrasound indices during the follow-up period was analyzed by Spearman’s correlation test. Baseline left ventricular internal diameter at end-diastole (LVIDd), left ventricular internal diameter at end-systole, and left ventricular ejection fraction (LVEF) were control variables to expel their influence on the correlation coefficients. When comparing the efficacy of hs-cTn-I and BNP in predicting the occurrence of endpoint events in idiopathic DCM patients, a receiver operating characteristic (ROC) curve was employed. The Hanley & McNeil test was used as the method for ROC comparison. We calculated the Youden index (sensitivity + specificity – 1) for each coordinate point in the ROC curve and selected the value of the biomarker corresponding to the maximum Youden index as the cut-off value. Net reclassification index (NRI) was used to compare the prognostic value between hs-cTn-I and BNP. All tests were bilateral tests, and *P* < 0.05 was considered statistically significant.

## 3 Results

### 3.1 General clinical features

The median follow-up period was 18 months (8–18 months). Of 122 patients with idiopathic DCM, 32 patients were readmitted due to heart failure, 20 patients had emergency visits due to heart failure, 2 had new-onset atrial fibrillation, and 1 died due to heart failure caused by DCM during the follow-up period. Table 1 summarizes the baseline characteristics between the non-end-event group (*n* = 67) and the end-event group (*n* = 55). Compared with the non-end-event group, the end-event group presented higher serum concentrations of cardiac troponin-I (median: 0.082 vs 0.026 ng/mL, *P* < 0.01) and BNP (median: 770 vs 424 pg/mL, *P* < 0.01). Other characteristics were comparable between the two groups (all *P* > 0.05).

**Table 1:** Baseline data of the non-end-event group and the end-event group

	Non-end-event group (*n* = 67)^a^	End-event group (*n* = 55)^a^	*P* value
**General characteristics**			
Male (%)	52 (77.61%)	41 (74.55%)	0.83
Age (year)	54.82 ± 12.9	58.64 ± 11.85	0.09
BMI^b^ (kg/m^2^)	25.71 (23.88, 29.07)	25 (23, 27.46)	0.12
Hypertension (%)	27 (40.3%)	18 (32.73%)	0.45
Diabetes mellitus (%)	11 (16.42%)	13 (23.64%)	0.37
Healthy dietary habits^c^ (%)	37 (55.22%)	29 (52.73%)	0.86
Sufficient exercise^d^ (%)	26 (38.81%)	14 (25.45%)	0.13
Daily salt intake (g)	6.19 ± 0.97	6.17 ± 0.93	0.94
**Laboratory test**			
hs-cTn-I^b^ (ng/mL)	0.026 (0.015, 0.058)	0.082 (0.047, 0.202)	<0.01^e^
BNP^b^ (pg/mL)	424 (146, 804)	770 (612, 1715)	<0.01^e^
CK-MB^b^ (ng/mL)	1.7 (0.9, 2.3)	1.8 (1.1, 3)	0.68
Myoglobin (ng/mL)	63.9 (37.2, 78.2)	74.6 (57.4, 91.3)	0.08
WBC^b^ (10^9^/L)	6.5 (5.2, 8.3)	6.73 (5.53, 8.05)	0.51
RBC^b^ (10^12^/L)	4.61 ± 0.67	4.63 ± 0.86	0.92
HB^b^ (g/L)	137.94 ± 20.32	140.56 ± 24.44	0.52
Hematocrit (%)	40.8 (37.6, 45.4)	42.1 (36.4, 46.7)	0.61
MCV^b^ (fL)	89.5 (87.1, 92.8)	89.9 (86.1, 93.8)	0.83
MCH^b^ (pg)	30.2 (29.4, 31.3)	30.2 (28.9, 31.9)	0.72
MCHC^b^ (g/L)	334 (326, 342)	334 (325, 343)	0.74
RDW^b^ SD (fl)	44 (40, 47.4)	42.8 (40.6, 47.3)	0.78
RDW^b^ variability (%)	13.3 (12.6, 14.7)	13.2 (12.4, 14.4)	0.7
PLT^b^ (10^9^/L)	206.84 ± 62.99	213.84 ± 75.82	0.58
CRP^b^ (mg/L)	3.61 (0.01, 15.47)	5.78 (1.26, 20.5)	0.12
INR^b^	1.11 (1.01, 1.22)	1.15 (1.05, 1.34)	0.07
APTT^b^ (s)	27.5 (25.5, 30)	28 (25.3, 30.6)	0.75
Fibrinogen (g/L)	2.98 (2.47, 3.41)	3.04 (2.55, 3.45)	0.59
D-dimer (ng/mL)	330 (190, 710)	590 (210, 1140)	0.14
ALT^b^ (U/L)	26 (15.3, 42.4)	25.8 (13, 49)	0.9
AST^b^ (U/L)	22 (17.4, 30)	23.6 (16, 37.3)	0.65
Triglyceride (mmol/L)	1.1 (0.81, 1.58)	1.12 (0.88, 1.38)	0.65
Total cholesterol (mmol/L)	4.24 (3.65, 5.05)	4.37 (3.54, 4.75)	0.59
HDL-C^b^ (mmol/L)	1.17 ± 0.35	1.1 ± 0.35	0.26
LDL-C^b^ (mmol/L)	2.55 (2.11, 3.28)	2.64 (2.17, 3.15)	0.96
Lipoprotein A (mg/L)	182 (131, 261)	150 (101, 235)	0.1
Potassium ion (mmol/L)	4.12 ± 0.44	4.07 ± 0.6	0.64
Sodium ion (mmol/L)	141 (139, 143.4)	141 (139, 143)	0.88
TSH^b^ (mIU/L)	2.21 (1.49, 2.54)	2.05 (1.2, 2.58)	0.21
FT3^b^ (pmol/L)	3.71 (3.43, 4)	3.72 (3.13, 3.96)	0.32
FT4^b^ (pmol/L)	15.18 (14.38, 16.37)	14.75 (13.67, 16.1)	0.12
eGFR^b^ (mL/min)	69.96 (59.6, 92.18)	64.49 (50.87, 86.21)	0.07
**Cardiac ultrasound indices**			
LVIDd^b^ (cm)	6.21 ± 0.83	6.47 ± 0.82	0.09
LVIDs^b^ (cm)	5.16 ± 0.93	5.37 ± 0.92	0.21
LVEF^b^ (%)	32 (28, 39)	34 (29, 40)	0.74

^a^Data are presented as the mean ± SD, *Q*_2_ (*Q*_1_, *Q*_3_) or *n* (%).

^b^BMI, body mass index; hs-cTn-I, high-sensitivity cardiac troponin I; BNP, brain natriuretic peptide; CK-MB, creatine kinase, MB form; WBC, white blood cell; RBC, red blood cell; HB, hemoglobin; MCV, mean corpusular volume; MCH, mean corpuscular hemoglobin; MCHC, mean corpuscular hemoglobin concentration; RDW, red blood cell distribution width; PLT, platelet; CRP, C-reactive protein; INR, international normalized ratio; APTT, activated partial thromboplastin time; ALT, alanine aminotransferase; AST, aspartate aminotransferase; HDL-C, high-density lipoprotein cholesterol; LDL-C, low-density lipoprotein cholesterol; TSH, thyroid stimulating hormone; FT3, free triiodothyronine; FT4, free tetraiodothronine; eGFR, estimate glomerular filtration rate; LVIDd, left ventricular internal diameter at end-diastolic; LVIDs, left ventricular internal diameter at end-systole; LVEF, left ventricular ejection fraction.

^c^More than four servings of fruits or vegetables per day and a daily fat intake less than 80 g and a daily free sugar intake less than 25 g.

^d^Either 30 min of moderate to vigorous physical activity (MVPA) on 5 days of the week and/or 30 min of vigorous physical activity three or more times per week.

^e^ *P* < 0.05, which means statistically significant.

### 3.2 Cox regression analysis of the correlation between clinical features and long-term prognosis in patients with idiopathic DCM

**Figure 1:**
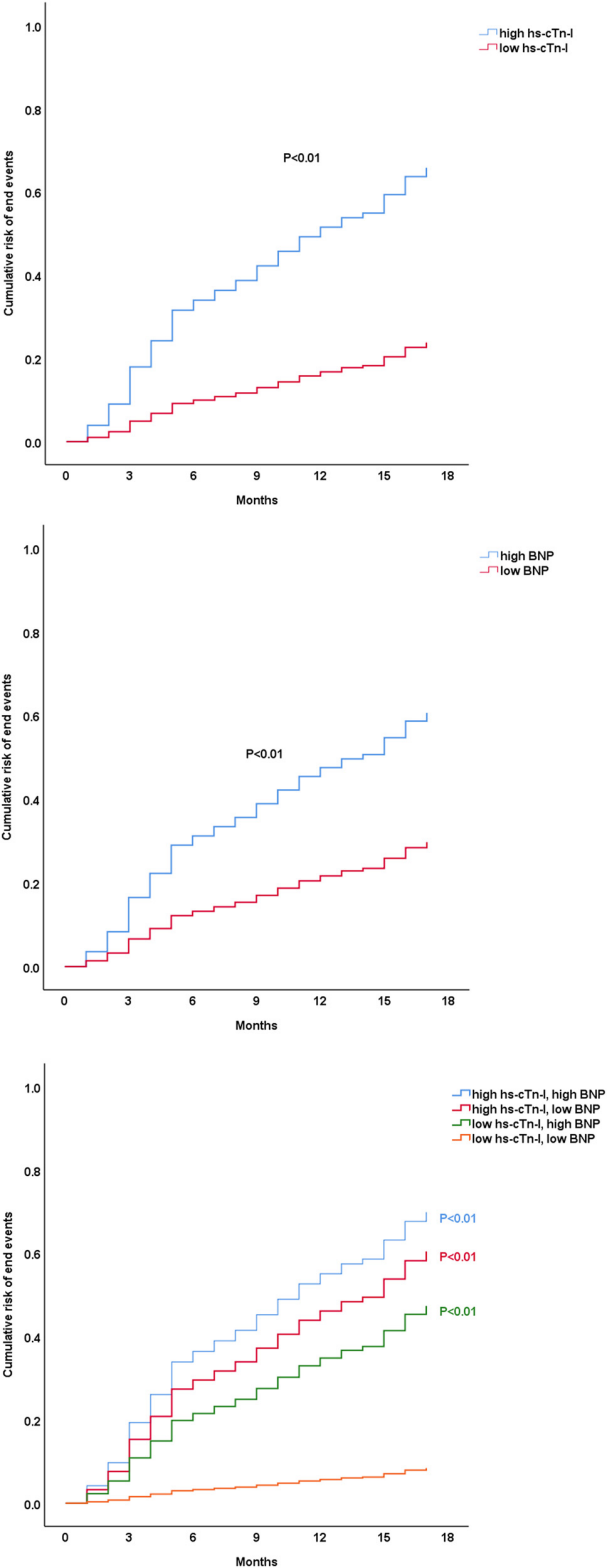
Survival curve for patients with different high-sensitivity cardiac troponin I and brain natriuretic peptide levels.

The factors associated with long-term outcome were analyzed by the multivariate Cox model. Table 2 demonstrates that high hs-cTn-I (HR = 5.91, 95% CI: 1.2–29.08, *P* value = 0.03) and high BNP (HR = 1.02, 95% CI: 1.01–1.03, *P* value < 0.01) were associated with an increased risk of poor prognosis in patients with idiopathic DCM. Patients with high hs-cTn-I and/or BNP levels had a higher incidence of endpoint events than patients with low hs-cTn-I and BNP levels, according to the survival curve (Figure 1a–c).

**Table 2:** COX regression analysis of risk factors related to end point events

	Univariate		Multivariate		
	HR	*P* value	HR	95% CI	*P* value
Male	0.86	0.62			
Age	1.02	0.06^d^	1.01	0.99–1.04	0.34
BMI^a^	0.95	0.11			
Hypertension	0.76	0.33			
Diabetes mellitus	1.45	0.24			
Healthy dietary habits^b^	0.92	0.77			
Sufficient exercise^c^	0.62	0.12			
Daily salt intake	1.02	0.9			
hs-cTn-I^a^	5.69	0.01^d^	5.91	1.2–29.08	0.03^e^
BNP^a^	1.02	<0.01^d^	1.02	1.01–1.03	<0.01^e^
CK-MB^a^	0.98	0.65			
Myoglobin	1	0.63			
WBC^a^	1	0.93			
RBC^a^	0.96	0.84			
HB^a^	1	0.77			
Hematocrit	1	0.53			
MCV^a^	0.99	0.58			
MCH^a^	1.02	0.21			
MCHC^a^	1	0.15			
RDW^a^ SD	1.03	0.18			
RDW^a^ variability	0.99	0.47			
PLT^a^	1	0.7			
CRP^a^	1.01	0.07^d^	1.01	0.99–1.03	0.17
INR^a^	2.42	0.05^d^	0.93	0.36–2.4	0.87
APTT^a^	1.02	0.62			
Fibrinogen	1.06	0.7			
D-dimer	1	0.46			
ALT^a^	1	0.68			
AST^a^	1	0.63			
Triglyceride	0.68	0.12			
Total cholesterol	0.88	0.3			
HDL-C^a^	0.65	0.28			
LDL-C^a^	0.95	0.74			
Lipoprotein A	1	0.86			
Potassium ion	0.8	0.42			
Sodium ion	0.97	0.47			
TSH^a^	0.89	0.3			
FT3^a^	0.73	0.1			
FT4^a^	0.94	0.28			
eGFR^a^	0.99	0.08^d^	0.99	0.99–1.01	0.92
LVIDd^a^	1.28	0.13			
LVIDs^a^	1.18	0.27			
LVEF^a^	1.01	0.51			

^a^BMI, body mass index; hs-cTn-I, high-sensitivity cardiac troponin I; BNP, brain natriuretic peptide; CK-MB, creatine kinase, MB form; WBC, white blood cell; RBC, red blood cell; HB, hemoglobin; MCV, mean corpusular volume; MCH, mean corpuscular hemoglobin; MCHC, mean corpuscular hemoglobin concentration; RDW, red blood cell distribution width; PLT, platelet; CRP, C-reactive protein; INR, international normalized ratio; APTT, activated partial thromboplastin time; ALT, alanine aminotransferase; AST, aspartate aminotransferase; HDL-C, high-density lipoprotein cholesterol; LDL-C, low-density lipoprotein cholesterol; TSH, thyroid stimulating hormone; FT3, free triiodothyronine; FT4, free tetraiodothronine; eGFR, estimate glomerular filtration rate; LVIDd, left ventricular internal diameter at end-diastolic; LVIDs, left ventricular internal diameter at end-systole; LVEF, left ventricular ejection fraction.

^b^More than four servings of fruits or vegetables per day and a daily fat intake less than 80 g and a daily free sugar intake less than 25 g.

^c^Either 30 min of moderate to vigorous physical activity (MVPA) on 5 days of the week and/or 30 min of vigorous physical activity three or more times per week.

^d^ *P* < 0.1, which means that variable was considered for inclusion in the multivariate model.

^e^ *P* < 0.05, which means statistically significant in the multivariate model.

### 3.3 ROC analysis of the prognostic value of hs-cTn-I in patients with idiopathic DCM

**Figure 2:**
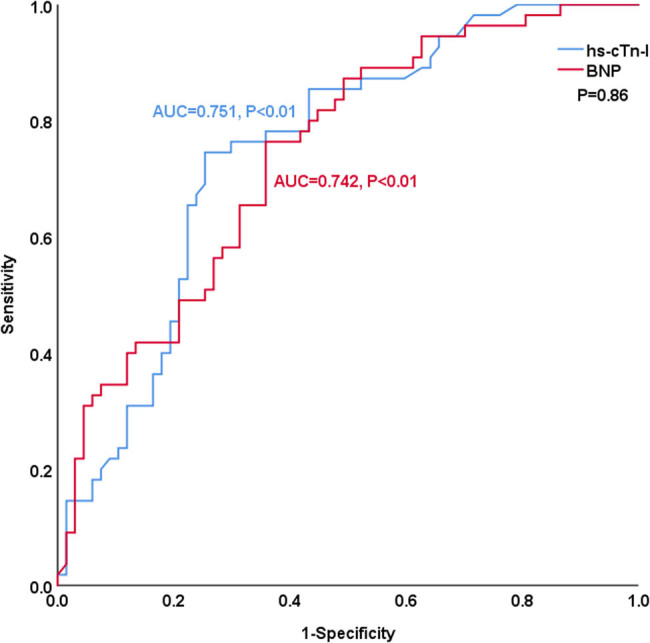
ROC analysis of the prognostic value of hs-cTn-I and BNP in patients with idiopathic dilated cardiomyopathy.

Figure 2 shows the comparison of the predictive ability of hs-cTn-I and BNP for endpoint events by ROC analysis. The areas under the curve (AUCs) of hs-cTn-I and BNP for predicting endpoint events were comparable (0.751 vs 0.742, *P* = 0.86, NRI = 0.086). The best cut-off value of hs-cTn-I to predict long-term prognosis by ROC analysis was 0.053 ng/mL (sensitivity: 74.5%, specificity: 74.6%). The best cut-off value of BNP to predict long-term prognosis by ROC analysis was 607.5 pg/mL (sensitivity: 76.4%, specificity: 64.2%).

### 3.4 Correlation among hs-cTn-I, BNP, and percentage change in cardiac ultrasound indices

Table 3 shows the correlations between hs-cTn-I and BNP levels and percentage changes in cardiac ultrasound indices during the follow-up period. hs-cTn-I was correlated with the percentage change in LVEF (correlation coefficient = −0.21, *P* value = 0.03) and the percentage change in LVIDd (correlation coefficient = 0.2, *P* value = 0.02), and BNP was correlated with the percentage change in LVEF (correlation coefficient = −0.21, *P* value = 0.02). These results indicate that high hs-cTn-I and BNP may be related to the deterioration of cardiac ultrasound indicators in idiopathic DCM patients.

**Table 3:** Correlation of the degree of high-sensitivity cardiac troponin I, brain natriuretic peptide and percentage change in cardiac ultrasound indexes

	hs-cTn-I^a^		BNP^a^	
	Correlation coefficient	*P* value	Correlation coefficient	*P* value
Percentage change in LVIDd^a^	0.2	0.03^b^	−0.01	0.99
Percentage change in LVIDs^a^	0.09	0.34	0.07	0.46
Percentage change in LVEF^a^	−0.21	0.03^b^	−0.22	0.02^b^

^a^hs-cTn-I, high-sensitivity cardiac troponin I; BNP, brain natriuretic peptide; LVIDd, left ventricular internal diameter at end-diastolic; LVIDs, left ventricular internal diameter at end-systole; LVEF, left ventricular ejection fraction.

^b^ *P* < 0.05, which means statistically significant.

### 3.5 Subgroup analysis

**Figure 3:**
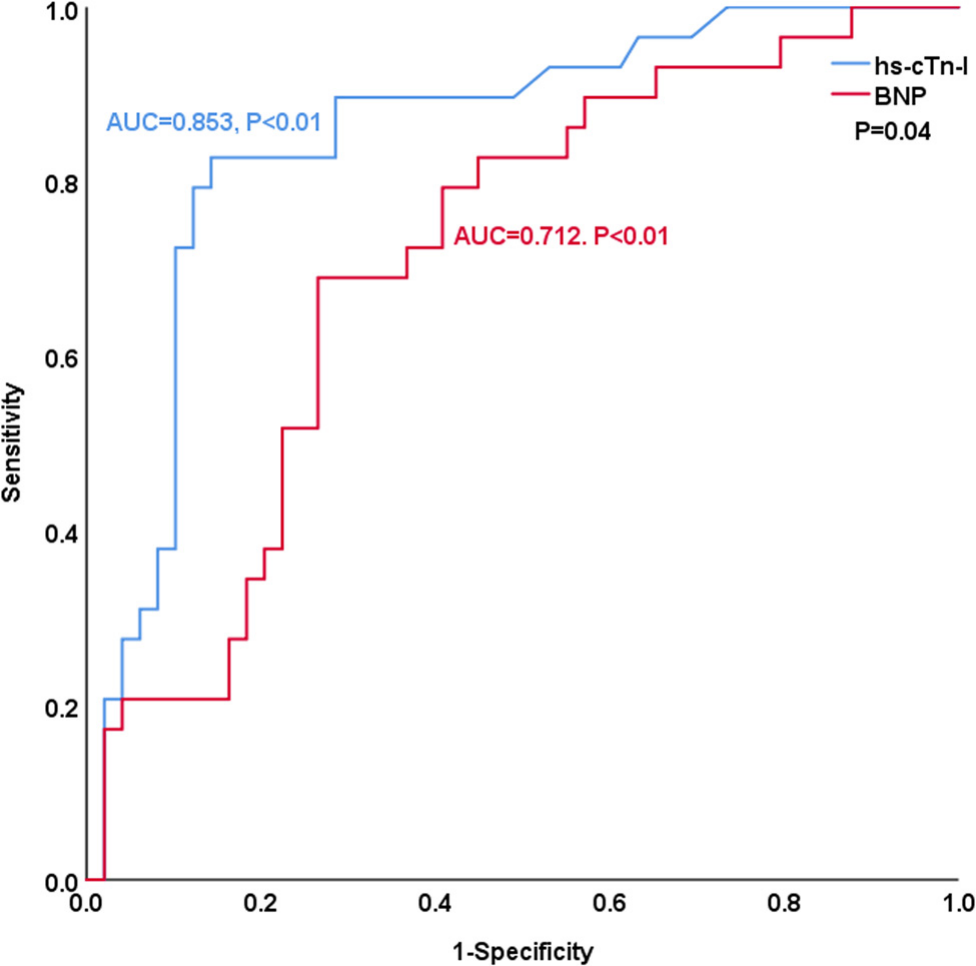
ROC analysis of the prognostic value of hs-cTn-I and BNP in idiopathic dilated cardiomyopathy patients with preserved renal function.

**Figure 4:**
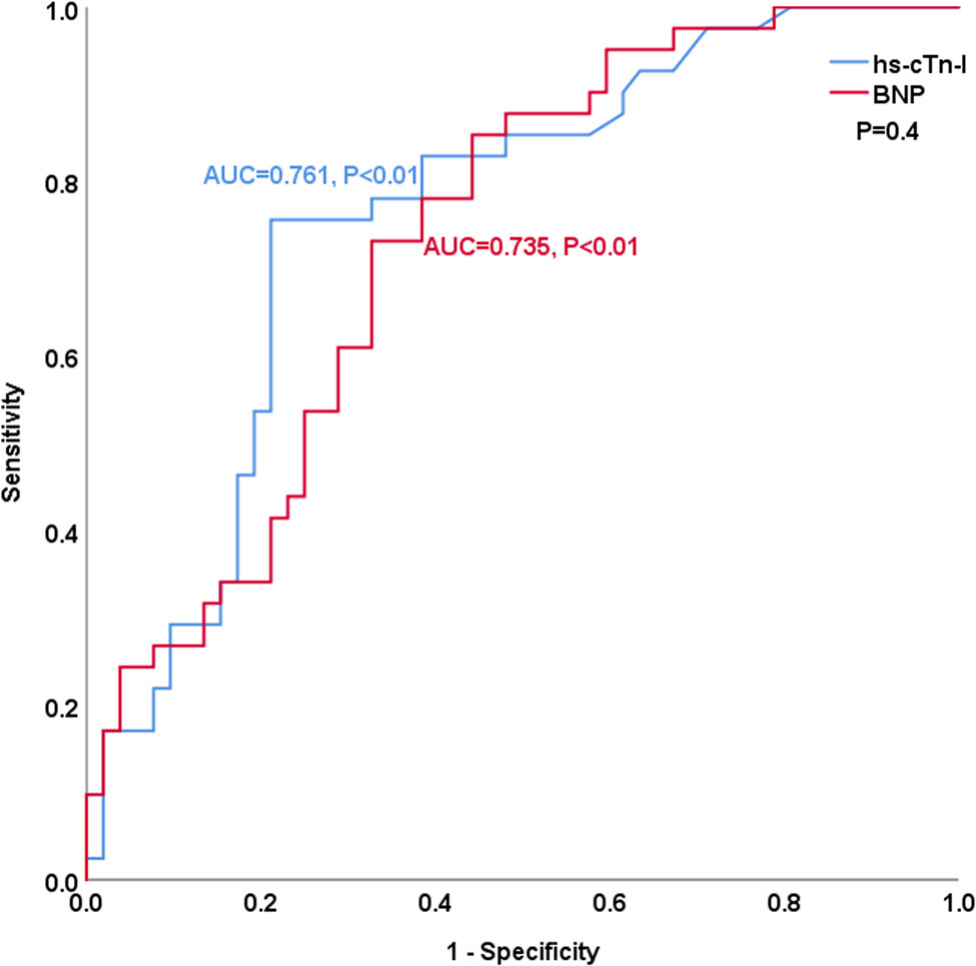
ROC analysis of the prognostic value of hs-cTn-I and BNP in males with idiopathic dilated cardiomyopathy.

**Figure 5:**
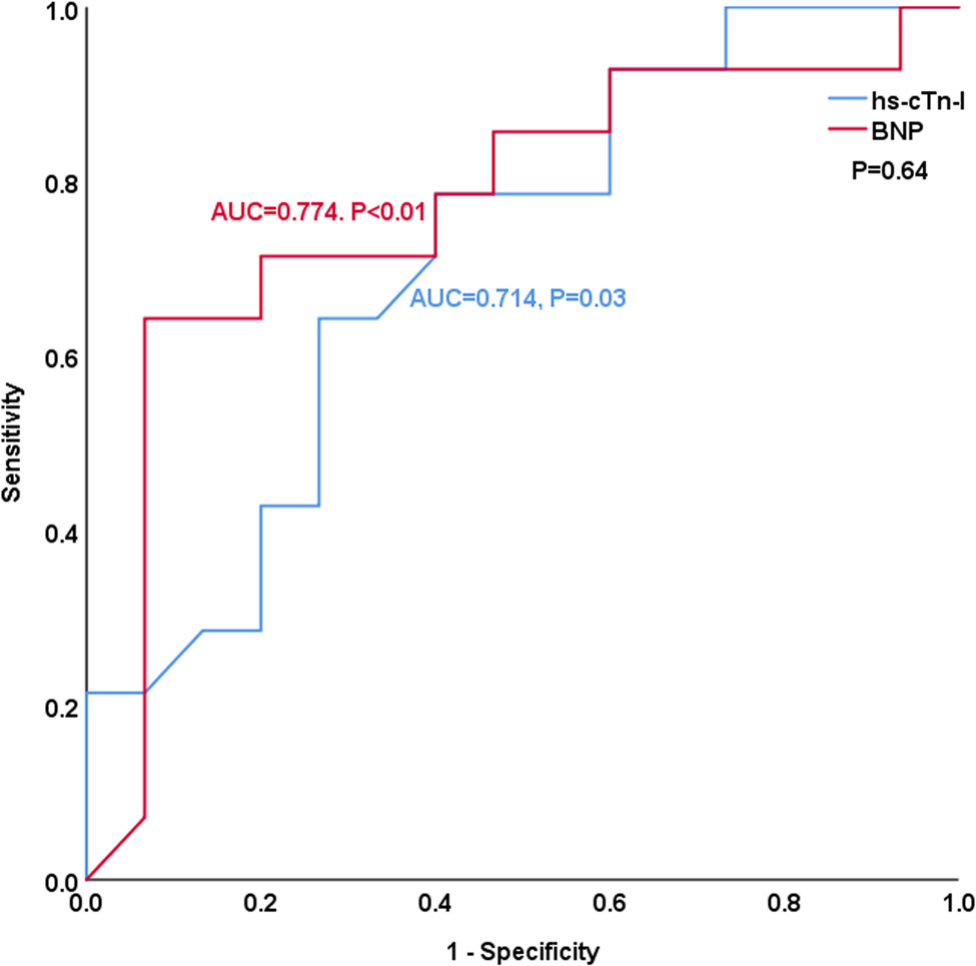
ROC analysis of the prognostic value of hs-cTn-I and BNP in females with idiopathic dilated cardiomyopathy.

**Figure 6:**
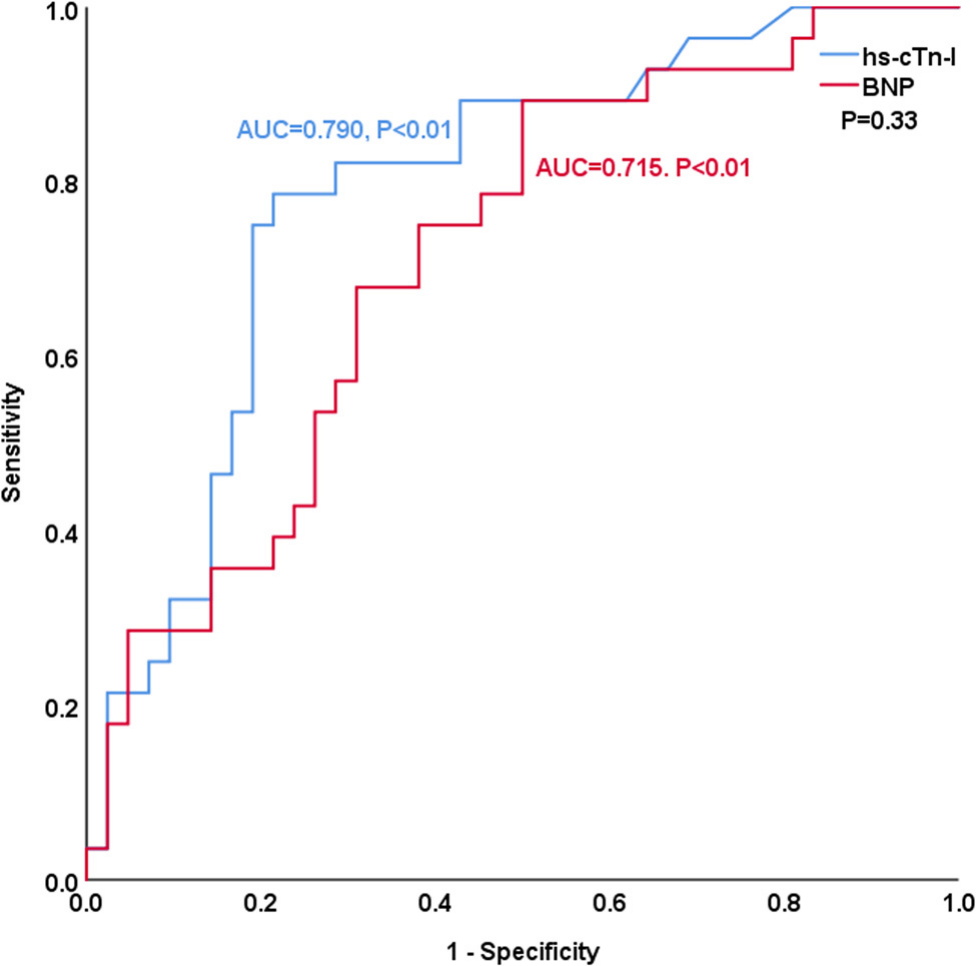
ROC analysis of the prognostic value of hs-cTn-I and BNP in idiopathic dilated cardiomyopathy patients with age less than 60 years.

**Figure 7:**
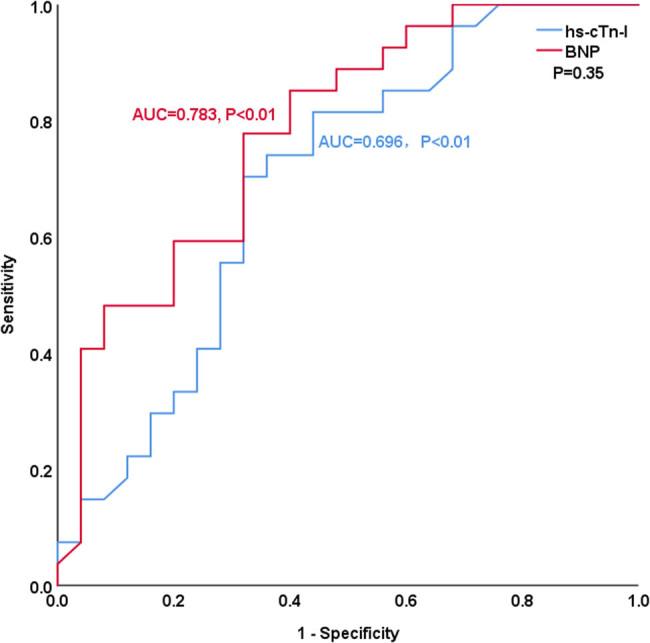
ROC analysis of the prognostic value of hs-cTn-I and BNP in idiopathic dilated cardiomyopathy patients with age equal or more than 60 years.

**Figure 8:**
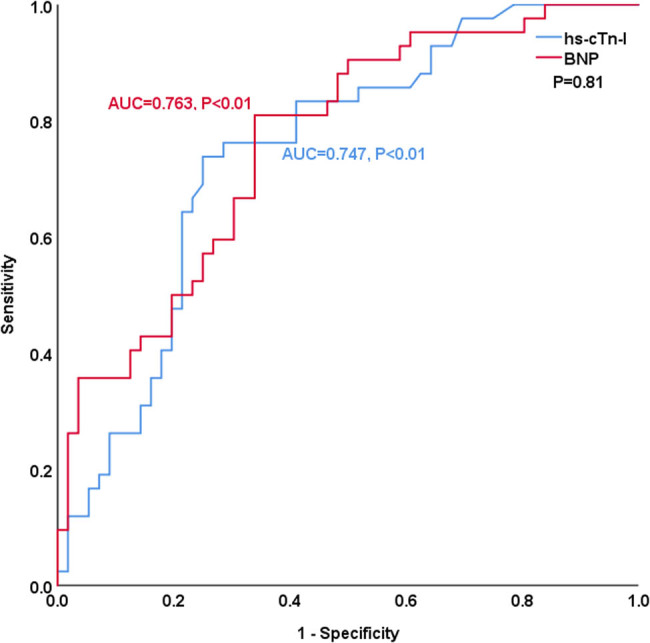
ROC analysis of the prognostic value of hs-cTn-I and BNP in idiopathic dilated cardiomyopathy patients without diabetes mellitus.

Table 4 and Figures 3–8 show the comparison of the predictive ability of hs-cTn-I and BNP for endpoint events in various subgroups by ROC analysis. Table 4 and Figure 3 show that the AUCs of hs-cTn-I and BNP for predicting endpoint events in patients with preserved renal function (estimated glomerular filtration rate >60 mL/min) were 0.853 vs 0.712, *P* = 0.04, NRI = 0.26, indicating that, compared with BNP, hs-cTn-I had a superior predictive value in patients with preserved renal function. The best cut-off value of hs-cTn-I to predict long-term prognosis by ROC analysis was 0.045 ng/mL (sensitivity: 82.8%, specificity: 85.7%). The best cut-off value of BNP to predict long-term prognosis by ROC analysis was 602.41 pg/mL (sensitivity: 69%, specificity: 73.5%). Table 4 and Figures 4–8 show that the AUCs of hs-cTn-I and BNP for predicting endpoint events in males, females, patients aged less than 60 years, patients aged 60 years or more, and patients without diabetes mellitus were comparable (all *P* > 0.05). Table 4 shows that the *P* values of hs-cTn-I for predicting endpoint events in idiopathic DCM patients with renal function insufficiency and the *P* values of BNP for predicting endpoint events in idiopathic DCM patients with diabetes mellitus were more than 0.05; consequently, we did not conduct further analysis on these two subgroups.

**Table 4:** The AUC of high-sensitivity cardiac troponin I and BNP for predicting endpoint events

	hs-cTn-I^a^		BNP^a^		Compare *P* value	NRI^a,d^
	AUC^a^	*P* value	AUC	*P* value		
All patients	0.751	<0.01^b^	0.742	<0.01^b^	0.86	0.086
Patients with eGFR^a^ >60 mL/min	0.853	<0.01^b^	0.712	<0.01^b^	0.04^b^	0.26
Patients with eGFR^a^ ≤ 60 mL/min	—	0.99^c^	—	—	—	—
Males	0.761	<0.01^b^	0.735	<0.01^b^	0.4	0.133
Females	0.714	0.03^b^	0.774	<0.01^b^	0.64	−0.191
Patients with age <60 years	0.79	<0.01^b^	0.715	<0.01^b^	0.33	0.179
Patients with age ≥60 years	0.696	<0.01^b^	0.783	<0.01^b^	0.35	−0.074
Patients with diabetes mellitus	—	—	—	0.2^c^	—	—
Patients without diabetes mellitus	0.747	<0.01^b^	0.763	<0.01^b^	0.81	0.018

^a^hs-cTn-I, high-sensitivity cardiac troponin I; BNP, brain natriuretic peptide; eGFR, estimate glomerular filtration rate; AUC, area under the curve; NRI, net reclassification.

^b^ *P* < 0.05, which means statistically significant.

^c^ *P* > 0.05, no further analysis was conducted.

^d^Brain natriuretic peptide as control.

## 4 Discussion

During the follow-up period of our study, the most common endpoint was readmission or an emergency visit due to heart failure (*n* = 52, 94.5%). BNP is the most common biomarker used for the diagnosis [5] and prognosis [6] of heart failure. In individuals with DCM, BNP has been demonstrated to predict the deterioration of LVEF [10]. Our study showed that patients in the end-event group tended to have higher hs-cTn-I and BNP levels. Therefore, we focused on calculating the prognostic value of hs-cTn-I in idiopathic DCM and compared it with that of BNP. The major findings from the present study are as follows. First, patients with poor outcomes had significantly elevated baseline hs-cTn-I and BNP levels, and high hs-cTn-I and BNP levels were associated with an increased risk of poor prognosis in patients with idiopathic DCM. Second, hs-cTn-I and BNP were potential prognostic biomarkers for long-term prognosis in patients with idiopathic DCM. Finally, compared with BNP, hs-cTn-I had a better predictive value for long-term prognosis in idiopathic DCM patients with preserved renal function.

Troponins are proteins that directly transmit the excitation of myocardial cells to cause contraction. When troponin-I maintains the stability of tropomyosin by binding actin and blocking the myosin binding site, relaxation of striated muscle occurs. When the conformation of the troponin complex changes due to Ca^2+^ influx, releasing troponin-I from the myosin binding site on actin, contraction of striated muscle occurs [11]. Cardiac troponin-I has unique expression in myocardial tissue, and it cannot be detected in developing or adult skeletal muscle samples. Therefore, degenerative muscle disease or muscle development can be excluded as the source of circulating cardiac troponin-I [12]. When the myocardium is damaged, the cardiac troponin-I levels in circulating blood change early and can be maintained for a long time, and therefore, such levels are a sensitive indicator of myocardial injury. Since its discovery, hs-cTn-I has played an important role in the diagnosis of acute myocardial infarction [13]. With the increasing sensitivity of detection methods, it should be emphasized that cardiac troponin-I is related not only to myocardial infarction but also to various cardiovascular diseases [14]. Studies have shown that hs-cTn-I can be used to evaluate the prognosis of patients with aortic stenosis [15], atrial fibrillation [16], and intracerebral hemorrhage [17]. Castiglione et al. [18] found that cardiac troponin-I was a potential diagnostic indicator for heart failure. Moreover, Xue et al. [19] found that elevated hs-cTn-I levels were linked to a poor prognosis in heart failure patients. In fact, the ACC/AHA guidelines advised using hs-cTn-I/T levels upon admission for risk stratification of heart failure (IA) [20], the endpoint event that occurred the most frequently in our study. According to an animal study [21], cardiac troponin-I is a factor in the diagnosis of DCM in Dobermanns. A clinical trial involving 310 patients with idiopathic DCM [3] revealed that in contrast to creatine kinase-MB (CK-MB) or myoglobin, serum cardiac troponin-I concentration was an independent predictor of all-cause mortality. However, the association between hs-cTn-I and cardiovascular events (e.g., cardiovascular death, heart failure) in patients with idiopathic DCM has rarely been reported. Our research showed that both hs-cTn-I and BNP were potential biomarkers for predicting the long-term prognosis of idiopathic DCM (AUC: 0.751, 0.742).

The following are possible explanations for the elevated hs-cTn-I in idiopathic DCM patients. Idiopathic DCM can lead to cardiac remodeling, which pathophysiologically manifests as a decrease in cardiac output, an increase in end-diastolic pressure, and an increase in cardiac preload. These changes lead to the activation of the renin–angiotensin–aldosterone system, an increase in sympathetic adrenergic activity, and a reduction in cardiac vagal activity [7]. The elevation of left ventricular end-diastolic pressure results in the release of cardiac troponin [22], and secondary neurohormonal changes lead to an increase in cardiac afterload, elevation of myocardial oxygen demand, and direct cardiotoxicity [23]. The combination of these changes injures myocardial cells, releasing cardiac troponin. There are also studies suggesting that the elevated cardiac troponin-I in patients with DCM is also related to immune-mediated dysfunction [24] or that a decline in energy supply results from the loss of healthy mitochondria in the heart [25]. In addition, elevated NLPR-3 has been reported to be associated with poor prognosis in patients with idiopathic DCM [26], and inhibition of NLRP-3 improves the prognosis of patients with DCM [27].

Nellessen et al. [28] demonstrated that persistent myocardial cell injury and ongoing cardiac troponin-I release are related to the onset and progression of chronic heart failure in patients with ischemic and nonischemic DCM. In patients with idiopathic DCM, the concentration of hs-cTn-I eventually stabilizes in the progressive and ongoing myocardial damage process that reflects the rate of myocardial injury caused by idiopathic DCM. In other words, patients with high concentrations of hs-cTn-I exhibit more severe myocardial damage caused by idiopathic DCM.

A clinical study [29] including 1,555 patients showed that renal function can influence the diagnostic and prognostic performance of hs-cTn-I. This may be because cTn-I is partially metabolized by the kidney, and thus, the hs-cTn-I levels in patients with poor renal function may be higher than those in patients with normal renal function. This partly explains why some patients with poor renal function and high hs-cTn-I levels did not have an end event during follow-up. To exclude the influence of renal function, patients with an estimated glomerular filtration rate >60 mL/min were included in the subgroup for analysis. Our results show that the AUCs of hs-cTn-I and BNP for predicting endpoint events in patients with preserved renal function were 0.853 vs 0.712, *P* = 0.04, indicating that hs-cTn-I had a superior predictive value compared with BNP. However, this finding needs to be further confirmed by studies with large samples. The *P* values of hs-cTn-I for predicting endpoint events in idiopathic DCM patients with renal function insufficiency and the *P* values of BNP for predicting endpoint events in idiopathic DCM patients with diabetes mellitus were more than 0.05. We believe that this is due to the small number of patients included in the subgroup analysis. Other subgroup analyses showed that the AUCs of hs-cTn-I and BNP for predicting endpoint events in patients with idiopathic DCM were comparable.

In the early stage of idiopathic DCM, the myocardial injury is too mild to be found by routine auxiliary examination, such as ECG and cardiac ultrasound. An animal study [30] showed that only 40 mg of myocardial necrosis is sufficient to increase the serum concentrations of cardiac troponin above the 99th percentile, which means that hs-cTn-I can be detected in the early stage of myocardial injury caused by idiopathic DCM. A recent study [31] found that late gadolinium enhancement is valuable for identifying high-risk patients with DCM, but due to the complexity of this method, it is difficult to promote in primary medical institutions. hs-cTn-I concentration detection is a simple laboratory test that can be performed in most medical institutions. The results of this study suggest that hs-cTn-I can be used to evaluate the long-term prognosis of patients with idiopathic DCM. According to our research findings, if hs-cTn-I is measured in patients with idiopathic DCM after their diagnosis, it can identify those patients who are at high risk of a poor prognosis and enable more aggressive treatment to improve their prognosis.

This study has some limitations. First, it is a retrospective and observational study, which may be affected by some confounding factors. Although we analyzed several factors that may influence prognosis, unmeasured factors could not be analyzed. Second, this study only performed one measurement of troponin and did not monitor the levels of troponin or other biomarkers during the follow-up period. Third, the diagnosis of idiopathic DCM was made by excluding common causes of secondary DCM, such as myocarditis, hypertension, valve disease, and ischemic heart disease, without an endomyocardial biopsy to support the diagnosis.

## 5 Conclusion

Based on whether the patient had an end event within the follow-up period, we divided patients initially diagnosed with idiopathic DCM into an end-event group and a non-end-event group. Patients in the end-event group had higher hs-cTn-I and BNP levels. Cox regression analysis suggested that hs-cTn-I and BNP were risk factors for poor long-term prognosis in patients with idiopathic DCM. The correlation analysis suggested that hs-cTn-I was related to the decrease in LVEF and the increase in LVIDd in patients with idiopathic DCM, while BNP was related to the decrease in LVEF. According to the results of the ROC analysis, both hs-cTn-I and BNP are potential biomarkers for predicting the long-term prognosis of idiopathic DCM. Moreover, compared with BNP, hs-cTn-I has a superior predictive value in patients with preserved renal function.
